# Phosphoinositides in the Hepatitis C Virus Life Cycle

**DOI:** 10.3390/v4102340

**Published:** 2012-10-22

**Authors:** Bryan Bishé, Gulam Syed, Aleem Siddiqui

**Affiliations:** 1 Division of Biological Sciences, University of California, San Diego. 9500 Gilman Dr., San Diego, CA, 92093, USA; Email: bbishe@ucsd.edu; 2 Division of Infectious Diseases, University of California, San Diego. 9500 Gilman Dr., San Diego, CA, 92093, USA; Email: gsyed@ucsd.edu

**Keywords:** HCV, hepatitis C, PI4P, phosphoinositides, PI4KIIIα, PI4KIIIβ

## Abstract

Eukaryotes possess seven different phosphoinositides (PIPs) that help form the unique signatures of various intracellular membranes. PIPs serve as docking sites for the recruitment of specific proteins to mediate membrane alterations and integrate various signaling cascades. The spatio-temporal regulation of PI kinases and phosphatases generates distinct intracellular hubs of PIP signaling. Hepatitis C virus (HCV), like other plus-strand RNA viruses, promotes the rearrangement of intracellular membranes to assemble viral replication complexes. HCV stimulates enrichment of phosphatidylinositol 4-phosphate (PI4P) pools near endoplasmic reticulum (ER) sites by activating PI4KIIIα, the kinase responsible for generation of ER-specific PI4P pools. Inhibition of PI4KIIIα abrogates HCV replication. PI4P, the most abundant phosphoinositide, predominantly localizes to the Golgi and plays central roles in Golgi secretory functions by recruiting effector proteins involved in transport vesicle generation. The PI4P effector proteins also include the lipid-transfer and structural proteins such as ceramide transfer protein (CERT), oxysterol binding protein (OSBP) and Golgi phosphoprotein 3 (GOLPH3) that help maintain Golgi-membrane composition and structure. Depletion of Golgi-specific PI4P pools by silencing PI4KIIIβ, expression of dominant negative CERT and OSBP mutants, or silencing GOLPH3 perturb HCV secretion. In this review we highlight the role of PIPs and specifically PI4P in the HCV life cycle.

## 1. Introduction

Hepatitis C virus (HCV) infection is a looming silent pandemic with an estimated 2%–3% of global population afflicted [1]. About 70% of infected individuals develop chronic hepatitis C which often progresses towards liver diseases like fibrosis, cirrhosis and hepatocellular carcinoma [1]. HCV infection is the leading indication for liver transplantation in the USA [2]. The current therapeutic regimen of pegylated interferon and ribavirin is often poorly tolerated, and less than fully effective against HCV genotype 1, a genotype predominant in Northern America and Europe. Two inhibitors of viral protease NS3, telaprevir and boceprevir, have been recently approved and many more direct-acting antivirals are in various phases of development. Although direct-acting antivirals are very effective, the rapid emergence of resistant mutants can lead to recurrence of infection, underscoring the need for the development of treatment strategies that target host factors critical for viral lifecycle. 

A member of the *Flaviviridae* family of viruses, HCV contains a positive-stranded RNA genome that encodes a ~3,000 amino acid polyprotein. This polyprotein is cleaved co- and post-translationally by cellular and viral proteases to form the viral proteome of three structural and seven non-structural proteins [3]. HCV replication, like that of other positive-stranded RNA viruses, occurs on endoplasmic reticulum (ER)-derived modified membranous structures termed “membranous webs” [4]. HCV modulates host lipid metabolism, leading to the enrichment of intracellular lipids, which can facilitate membrane fluidity and availability, as well as the accumulation of lipid droplets (LDs) critical for assembly of viral replication complexes and morphogenesis respectively [5,6,7]. Viral replication complexes are often localized adjacent to LDs, which subsequently serve as platforms for post-replicative events of viral assembly and maturation [5]. Although not completely understood and not universally agreed upon, the prevailing view is that viral particles exit the cell by co-opting the very low-density lipoprotein (VLDL) secretion pathway, which is quite distinct from typical cellular protein secretion [8,9,10,11,12]. In the quest for identifying novel host factors critical for the HCV lifecycle, studies applying genome wide or targeted siRNA screens have identified the phosphatidylinositol-4-phosphate (PI4P) pathway as pivotal element in HCV replication [13,14,15,16,17].

**Figure 1:**
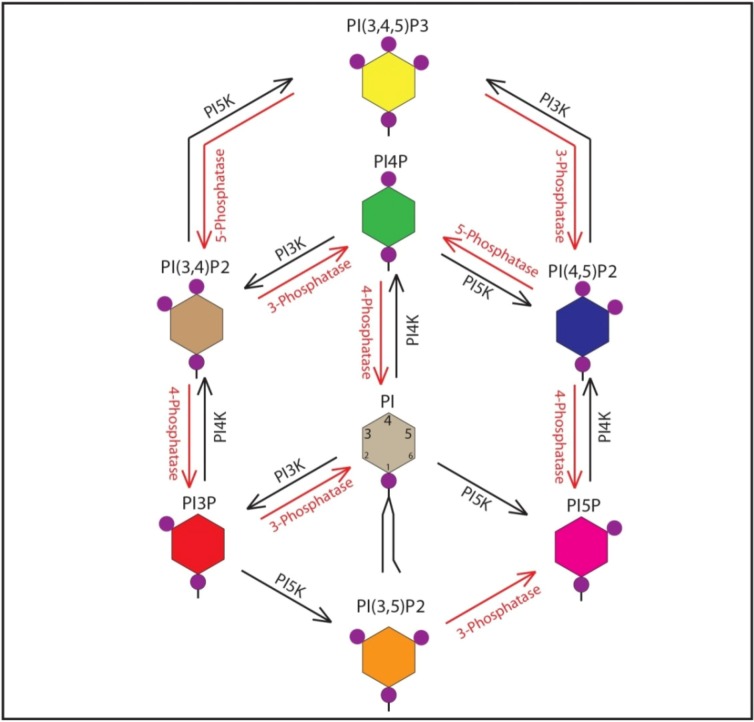
Phosphoinositides (PIPs), Kinases and Phosphatases. Schematic representation of phosphatidylinositol, the seven PIP varieties, kinases and phosphatases involved. Kinases are represented in black, phosphatases in red.

Phosphoinositides (PIPs) are phosphorylated derivatives of phosphatidylinositol (PI), an essential phospholipid component of the eukaryotic cell membrane [18]. PI can be mono-, di-, or tri-phosphorylated at the D-3, D-4 and D-5 positions of the inositol ring in various combinations to generate seven different phosphoinositides, which have distinct biological activity (Figure 1) [18]. A cohort of cellular phosphoinositide kinases and phosphatases control the interconversion of phosphoinositide species. So far 18 phosphoinositide interconversion reactions mediated by 19 phosphoinositide kinases and 28 phosphoinositide phosphatases have been identified [19]. PIPs are predominantly localized on the cytosolic side of membranes and are fundamental constituents of the cytosol-membrane interface [20]. The distinct make up of PIPs within a membrane constitutes a lipid code or membrane signature that defines the identity of the membrane and associated organelles. These PIPs interact with various effector and adaptor proteins, which localize to specific organelles via their PIP interacting domains [21]. A number of recent studies have implicated PI4P and several interacting partners in HCV infection. In uninfected cells PI4P is primarily localized to the Golgi, with smaller distinct pools in the ER [22,23]. HCV infection robustly stimulates ER-specific PI4P pools by activating phosphatidylinositol(4)phosphate kinase IIIα (PI4KIIIα), responsible for generating ER-specific PI4P [24,25]. In addition, we have shown that PI4P pools at the Golgi, and related PI4P-binding proteins are crucial for HCV secretion [26]. In this review, we will summarize all the recent findings and observations that highlight the role of phosphoinositides, their kinases, and their multiple binding partners in HCV infection.

## 2. Cellular Functions of Phosphoinositides

**Figure 2:**
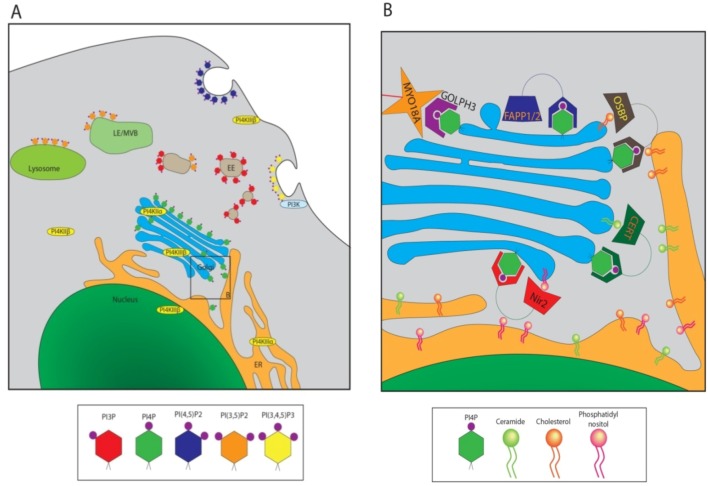
Cellular Localizations of Various Phosphoinositides. (**A**) Diagram of a typical cell, highlighting the locations of various PIPs in different cellular membranes. EE: Early endosome; LE/MVB: Late endosome/multi-vesicular body. Some PI kinases are also given in their cellular locations. PI4Ks are in yellow ovals, PI3K in cyan; (**B**) Close-up of panel B in Figure 2A. PI4P binding proteins at the Golgi/TGN are localized, as well as the lipids or other proteins to which they bind. Lipid transfer proteins (LTPs) Nir2, CERT, and OSBP are shown shuttling their preferred lipid cargo from the ER; PI (pink), ceramide (green), and cholesterol (orange), respectively.

Phosphoinositides serve two main functions in the cell; establishing membrane identity for various cellular organelles, and signaling [27]. Spatio-temporal modulation of a cohort of phosphoinositide kinases and phosphatases maintains distinct populations of PIPs that help define the membrane characteristics of different organelles. For instance, PI(4,5)P_2_ is located primarily at the plasma membrane (PM), whereas PI3P is predominantly an endosomal marker, and PI4P is present most frequently at the Golgi [22] (Figure 2A). These PIP populations in turn interact with proteins possessing binding domains specific for each phosphorylated derivative and thus serve as primary modulators of membrane-cytosol interactions and organelle identity [28,29]. For example specific PI4P binding pleckstrin homology (PH) domains shared by the lipid transfer proteins such as OSBP, CERT or four phosphate adaptor protein 2 (FAPP2) allow their localization to the Golgi [30] (Figure 2B). Most interactions between PIPs and their binding domains are weak, and require interactions with membrane proteins or membrane bound GTPases that often function as co-receptors with PIPs for the recruitment of cytosolic proteins to specific membrane compartments [31]. As mammalian cells express over 100 small GTPases, a wide variety of unique membrane domains can be established through dual interactions with specific GTPase and PIP [23,24]. Apart from the regulation of signal transduction at the membrane surface, the myriad functions of phosphoinositides include cytoskeleton and membrane reorganization, regulation of intracellular vesicular trafficking and Golgi secretory functions [20]. In addition, PIPs and their byproducts can function as members of signaling pathways [27]. The well-known secondary messengers, inositol 1,4,5-trisphosphate (IP_3_) and diacyl glycerol (DAG) are produced by phospholipase C (PLC) from PI(4,5)P_2_, where both components of the phosphoinositide function downstream to initiate a plethora of cellular responses.

PI4P, long considered merely an intermediary in the synthesis of PI(4,5)P_2_ for signaling at the plasma membrane, has recently garnered more attention as a number of studies have characterized its role in the maintenance of Golgi structure and function. PI4P is primarily produced by the monophosphorylation of PI at the D-4 position of the inositol ring (Figure 1) by one of four different phosphoinositide 4 kinases (PI4Ks) [32]. PI4P can also be produced via the dephosphorylation of PI(4,5)P_2_ by inositol polyphosphate-5-phosphatases [33]. PI4P is the most abundant monophosphorylated PIP in the cell, and the majority of PI4P localizes to the Golgi [34]. The Golgi has also been recognized for harboring highest levels of PI4K activity in the cell. PI4Ks have been categorized into two types: type II, which display sensitivity to adenosine, and type III, which are sensitive to Wortmannin [23]. Based on the domain structure there are two isoforms of each type of PI4K; PI4KIIα and PI4KIIβ; PI4KIIIα and PI4KIIIβ (also known as PI4Kβ). The Golgi is home to two types of PI4Ks; PI4KIIIβ, considered the kinase primarily responsible for establishing Golgi PI4P; and PI4KIIα, which has recently received attention due to a key role in the trans-Golgi (TGN) to plasma membrane (PM) transport [22,35]. These PI4Ks locate primarily at the Golgi, but the kinases can also be found in other membrane compartments in smaller amounts, namely in the endosomes, plasma membrane, and nucleus [22]. PI4KIIβ is mostly cytosolic, and can be recruited to the PM through activation by Rac1 [21]. PI4KIIIα is primarily located at the ER, and maintains a distinct, though smaller, population of PI4P which apparently contributes to the formation endoplasmic reticulum exit sites that regulate export from ER during acute and chronic increase in ER cargo load [36,37]. PI4KIIIα has also been shown to play a role in regulating PI4P levels at the PM [38,39]. hSac1 phosphatase is responsible for de-phosphorylation of PI4P with notable specificity [40]. Under normal conditions, hSac1 primarily localizes to the ER, though a small amount can be found in the ER-Golgi and Golgi compartments [40]. hSac1 localizes to the ER via its C-terminal K(*X*)K*XX* domain, and mutations to this structure cause hSac1 to accumulate at the Golgi rather than the ER [40,41].

A number of Golgi-specific cellular proteins localize to that compartment via their interactions with PI4P. Nir2, a lipid transfer protein (LTP) binds to PI4P, and is responsible for transfer of PI and phosphatidylcholine (PC) from the ER to the Golgi [42]. OSBP and CERT are important for lipid transport between the ER and Golgi for cholesterol and ceramide, respectively [43,44]. These three LTPs associate with the vesicle-associated membrane protein-associated proteins (VAPs) at the ER, and likely coordinate the majority of the lipid composition of the Golgi membranes by facilitating the formation of hypothetical ER-Golgi membrane contact sites by simultaneous interaction with PI4P in Golgi and VAP in ER [42]. Each of the proteins contains a PH binding domain, accounting for their association with PI4P. This binding is crucial for their localization to the Golgi compartment [43]. FAPP1 and FAPP2 localize to the TGN, also via a PH domain [21]. FAPP2 also plays a role in lipid transport, and is additionally important for Golgi-to-PM transport. A recently characterized PI4P-binding protein, GOLPH3 also binds PI4P through a binding pocket similar to a PH domain [41]. GOLPH3 interacts with an unconventional myosin, MYO18A, which in turn joins the TGN to the cellular actin cytoskeleton. The tensile force exerted on the Golgi by this interaction is thought to give the Golgi its flattened stack morphology, and is also important for vesicle budding from the TGN [41].

## 3. PI4P Significance in HCV Replication

**Figure 3:**
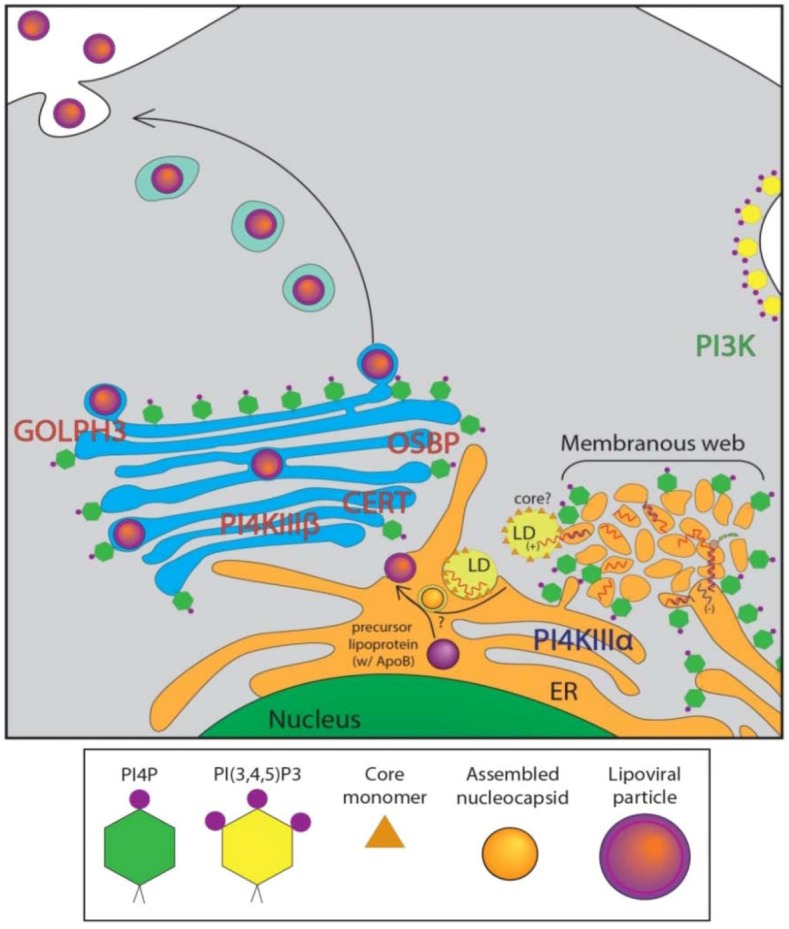
HCV Infected Cellular Distribution of PIPs, Binding Proteins. Diagram of HCV-infected cell showing enrichment of PI4P at the ER and membranous web, with model of potential HCV assembly pathway in conjunction with lipid droplets (LDs) and ApoB precursor lipoprotein [7]. HCV RNA is produced in replication complexes at membranous web structures juxtaposed to ER, and in close association with LDs. Viral assembly site is unknown, but viral particles may associate with ApoB precursor lipoproteins. Infectious viral particles are then likely trafficked through the Golgi and released at the PM. PIP associated proteins known to affect the HCV lifecycle are highlighted. Akt pathway-PI3K (green text). Replication-PI4KIIIα (blue text). Secretion-PI4KIIIβ, OSBP, CERT, GOLPH3 (red text).

Viruses are obligatory intracellular parasites and rely on host cell infrastructure for viability and proliferation. Positive-stranded RNA viruses are critically dependent on intracellular membrane rearrangement for RNA replication [45,46]. They modulate host lipid metabolism to influence the lipid composition of membranes, altering the membrane biophysical properties, possibly to facilitate membrane remodeling [47]. RNA viruses are known to utilize membranes of various organelles including ER, Golgi, trans-Golgi, endosomes and mitochondrial outer membrane to assemble replication platforms [48]. Many RNA viruses, including coxsackievirus, Aichi virus and enterovirus71 require enrichment of PI4P in replication membranes for active replication, which they achieve by the recruitment of host PI4Ks for the synthesis of PI4P from PI by various mechanisms [25,49,50,51]. In the case of HCV, studies utilizing siRNA-based screens have established the requirement of PI4Ks for HCV replication [13,14,15,16,17,24,52,53,54,55]. Liver biopsies from HCV patients also displayed high levels of PI4P, indicating that induction of PI4P is a clinical hallmark of HCV infection [24]. However, some discrepancies exist with regards to the role of specific PI4K isoforms, due to variations in experimental set up, analysis, and the HCV genotypes involved. Hsu *et al*. were the first to observe robust stimulation of ER-localized PI4P pools during HCV genotype 1b infection [25]. The typical Golgi/TGN localization of PI4P in uninfected cells was altered upon HCV infection to a discrete punctate distribution of PI4P in the cytoplasm [6,25,26]. Both PI4KIIIα and β were implicated in the induction of PI4P levels, and expression of hSac1 phosphatase in HCV infected cells both reduced PI4P levels and led to a decline in viral RNA synthesis [25]. Subsequent studies established the role of PI4KIIIα in membrane reorganization into replication competent membranous web-like structures [24,55]. These studies further strengthen the evidence for involvement of PI4KIIIα in HCV replication by revealing that HCV NS5A protein physically interacts and activates PI4KIIIα. NS5A domain 1 was shown to be required for recruitment and activation of PI4KIIIα. Deletion mutants of PI4KIIIα and NS5A map the interaction domain of PI4KIIIα to amino acids 401–600 and domain I of NS5A [56]. Abrogation of PI4KIIIα activity by chemical inhibitor PIK93 or siRNA-mediated gene silencing resulted in an aberrant clustering phenotype of NS5A in contrast to its normal reticular staining pattern [24]. Likewise, NS3 and NS5B immunostaining also showed aberrant clustering into large masses that entirely colocalized with NS5A, suggesting that silencing PI4KIIIα results in aggregation of viral replicase protein [24,54,55]. These studies indicate that NS5A recruits PI4KIIIα to the membranous replication compartment and stimulates PI4KIIIα activity resulting in robust induction of PI4P pools that are required to maintain the integrity of the membranous web structure (Figure 3). It has also been suggested that HCV alters the cellular distribution of PI4P, causing enrichment in the HCV-membranous web with a concomitant depletion from the PM and Golgi [57]. Despite the dramatic increase in cellular PI4P levels by HCV infection, the overall expression level of the PI4KIIIα is not significantly altered, suggesting that increased PI4P levels are the result of an increased kinase activity of PI4KIIIα [24]. Berger *et al*. also showed that the viral NS5A protein both recruits and activates PI4KIIIα, and that its activation is required for membranous web integrity as well as viral replication [55]. The significance of another PI4K isoform, PI4KIIIβ, normally localized to the trans-Golgi, in HCV replication is less clear [18,55]. PI4KIIIβ was never a top hit in several siRNA screens performed for cellular factors of HCV replication. PI4KIIIβ does not colocalize or interact with NS5A, and its overexpression does not rescue HCV replication hampered by PI4KIIIα silencing [24,54]. Interestingly, Hsu *et al.* reported that knockdown of PI4KIIIβ had a stronger effect on reduction of HCV genotype 1b replication than the knockdown of PI4KIIIα. This discrepancy could have been due to the use of only genotype 1b replicon in their assay. Tai and Salloum also implicated PI4KIIIβ in HCV replication but suggested the involvement of an alternative replication mechanism, since PI4KIIIβ knockdown did not affect membranous web formation or the accumulation of PI4P pools at these sites [54]. However, it should be noted that Tai *et al.* collected and assayed culture supernatants for *Gaussia* luciferase activity to assess the effect of PI4KIIIβ knockdown on HCV replication. PI4KIIIβ silencing is known to affect Golgi-secretory functions and hence may perturb secretion of the *Gaussia* luciferase reporter [58]. Zhang *et al.* also confirmed the requirement of the PI4KIIIβ for HCV genotype 2a replication and also noted the role of the small GTPase ARF1 and its guanine nucleotide exchange factor GBF1 [59]. ARF1 is known to recruit PI4KIIIβ to the TGN to stimulate PI4P production [60]. The role of PI4KIIIβ in HCV replication is still controversial, with several other groups finding evidence that PI4KIIIβ affects HCV secretion, but does not play a role in viral replication [26,55,61]. Further complicating the issue is the fact several replication systems and genotypes are in use, making comparison or consensus difficult.

Although the requirement of PI4P for HCV replication and integrity of the membranous web has been strongly established, the precise nature of this requirement still needs to be unraveled. In principle, HCV NS4B protein is capable of inducing membrane rearrangement [62,63]. Recent studies, however, implicate a formation of a more heterogeneous population of membrane structures involving double membrane vesicles and monomembrane vesicles, suggesting a more complex underlying mechanism [24,45,64]. The protein-lipid and protein-protein interactions might facilitate the membrane recruitment and confined enrichment and local concentration of viral proteins and critical host factors for establishment of functional replication complexes [65]. For instance, the RNA dependent RNA polymerase of poliovirus, a positive-sense RNA virus of the *Picornaviridae* family, shows high affinity for PI4P despite lacking any canonical PI4P binding domain [25]. The potential for HCV RNA-dependent RNA polymerase (RdRp) or NS5B to interact with PI4P still needs to be ascertained. PI4P could also help induce conformational alterations in its binding partners, such as viral replicase proteins, modulating their activity. PI4P localized at viral replication sites may help recruit cellular proteins that harbor PI4P binding domains such as PH domain-containing lipid transfer proteins (LTPs) like OSBP and CERT, which may facilitate the enrichment of cholesterol, sphingomyelin, and/or ceramide in replication compartments [42,66,67]. The dual interaction exhibited by such LTPs, with PI4P via their PH domains and with the ER by virtue of their FFAT motifs, may lead to the formation of membrane contact sites between replication membranes and the ER to sustain a supply of PI and lipids to the replication compartments. Indeed the role of OSBP and CERT in HCV replication has been already established and cholesterol-depleting agents such as β-cyclodextrins have been shown to disrupt the structure of the membranous web [63,64,66,68,69,70]. Incorporation of cholesterol in HCV replication membranes may alter the biophysical properties of the membrane and facilitate the assembly of HCV replication complexes [47]. PI is synthesized in the ER and delivered to other cellular locations via vesicular transport or by cytosolic PI transfer proteins such as Nir2 [20]. Exploring the role of Nir2 in HCV-mediated induction of PI4P in replication sites may prove useful. Since HCV replication complexes are closely associated with LDs that serve as platforms for viral assembly, the lipid alterations occurring at replication sties may also be evident in the viral envelope [5,71]. A recent report characterizing the lipidome of the viral particle shows characteristic enrichment of phosphatidylcholine, sphingomyelin, cholesterylester and cholesterol in HCV virus particle [72]. Some viruses have been shown to induce highly ordered complex structures dependent on cholesterol termed “cubic membranes,” that help sequester viral proteins and RNA for optimal replication and protect the viral components from host innate immune responses [47]. In addition, the local accumulation of anionic PI4P can also result in the alteration of the biophysical properties of membranes. A study with metabolically stabilized derivatives of PI4P that can substitute the natural lipid in protein recruitment and membrane deformation suggests that recruitment of proteins by PI4P is essential for inducing membrane tubulation [73]. This suggest that mere enrichment of PI4P pools is not sufficient to induce assembly of active HCV replication complexes but the recruitment of proteins by the PI4Ps is essential for inducing necessary membrane modifications. Identification of the precise function of PI4P and interacting viral or host proteins in assembling replication complexes could prove to be fundamentally important in designing novel anti-HCV therapeutics. 

Apart from the role of PI4Ks in HCV replication and secretion, their influence on HCV entry may suggest the importance of PI4Ks in multiple stages of HCV infection. Trotard *et al.* have reported that PI4KIIIα and β are also essential for HCV entry. However, they observed differential sensitivity of various HCV genotypes to the two PI4KIII isoforms suggesting that the two kinases regulate distinct events during HCV entry [52]. Silencing PI4KIIIα expression protected cells from HCVpp infection of genotype 1a whereas silencing PI4KIIIβ inhibited entry of HCVpp of genotype 1a and 1b [52]. The direct link of PI4Ks with the endocytic pathway is via PI4P that serves as precursor for PI(4,5)P_2_ synthesis at plasma membrane and is required for recruitment of adaptor proteins involved in clathrin-mediated endocytosis [74]. Interestingly, the PI4KIIIα and β knockdown only had a subtle effect on clathrin-mediated endocytosis suggesting that the effect of HCV entry is not due to inhibition of endocytosis [52]. Several other groups analyzing the effect of PI4Ks on HCV replication have not found any effect of PI4K knockdown on HCV entry, so this effect is also highly debated. HCV entry is a multi-step process involving complex interplay between various receptors and subsequent downstream signaling which may involve phosphoinositides other than PI4P, hence further investigations are needed to unravel the mechanistic details of the precise role of PI4Ks in HCV entry.

## 4. Role of Golgi-localized PI4P in HCV Secretion

In addition to the HCV-stimulated PI4P pools necessary for viral replication, there is growing evidence that PI4P and its binding proteins at the Golgi play a key role in viral secretion. HCV secretion has not been well defined, though it is thought to co-opt the very low-density lipoprotein (VLDL) secretion pathway [6,7,10,11,12,75]. VLDL is known to secrete through the Golgi compartment [9]. A number of recent studies have implicated the Golgi, and several PI4P binding proteins at the Golgi, in playing crucial roles in the viral secretion process. OSBP and CERT are both necessary for HCV secretion [68,69]. OSBP’s PH domain is critical for ensuring proper function of the protein at the Golgi and deletion or mutation of this domain abrogates HCV secretion [68]. Similarly, a CERT inhibitor dramatically curtailed HCV secretion [69] (Figure 3).

Distinct from a possible role in HCV replication, PI4KIIIβ and PI4Ps at the Golgi have recently been shown to play a role in the process of HCV secretion [26,61]. Silencing of PI4KIIIβ was found by Coller *et al.* to increase the ratio of intracellular to extracellular virus in HCV infected cells [61]. Essentially, PI4KIIIβ silencing promotes the retention of fully infectious viral particles within the cell, rather than their proper secretion. We confirmed this finding in our own study, and additionally showed that maintaining PI4P levels at the Golgi is also important for efficient HCV secretion [26]. We used a mutant version of hSac1, hSac1 K2A, which lacks the K(*X*)K*XX* domain required for the protein’s localization to the ER. Lacking this domain, hSac1 K2A localizes exclusively to the Golgi [40]. Expression of hSac1 K2A reduced the PI4P levels at the Golgi, and mimicked the effect of silencing PI4KIIIβ; intracellular viral infectivity increased while secretion of virus to the supernatant was decreased [26]. 

We also investigated the role of two additional proteins, GOLPH3 and MYO18A in HCV secretion. GOLPH3 binds to PI4P at the TGN, and also interacts with MYO18A [41]. These two proteins are responsible for tethering the TGN to the actin cytoskeleton, and are required for efficient budding of vesicles from the TGN. When their localization or binding abilities are abrogated, the Golgi loses its traditional flattened stack morphology, and vesicle budding is impaired. siRNA knockdown of either GOLPH3 or MYO18A had an effect on HCV secretion similar to hSac1 K2A expression, causing infectious HCV particles to be retained within the infected cell [26]. Taken together, these interactions point to a crucial role of a functioning Golgi network for proper viral secretion, but not for viral replication or assembly (Figure 3). Intriguingly, none of these strategies significantly altered VLDL secretion, raising some questions about the widely-held view of their association with HCV virion secretion. Another study of viral protein dynamics recently implicated several members of the Golgi secretory pathway of the cell in HCV secretion, as well as providing images of HCV core protein in transit [61]. An siRNA screen identified several cellular secretory proteins involved in Golgi structure and function or TGN to PM trafficking. These included PI4KIIIβ as well as protein kinase D 1 (PRKD1), adaptor-related protein complex 1 mu-1 (AP1M1), vesicle-associated membrane protein (VAMP1) and ras-related RAB11A. Using a TC-tagged HCV core protein, they were able to show microtubule-dependent movement of the core protein through the TGN. Brefeldin A (BFA) and PIK93, inhibitors of ARF3 and PI4KIIIβ, respectively, slowed this movement. While in transit, the tagged core protein colocalized with Apolipoprotein E, a VLDL component, and also associated with several components of the secretory pathway including Rab11a and VAMP1 [61]. These studies provide strong evidence for the trafficking of core protein through the Golgi.

## 5. HCV and PI3K Activation

PI4P is not the only PIP of significance in HCV infection. Several reports have indicated the activation of PI3K/Akt pathway in HCV infection and several downstream kinases like mTOR and S6K1 [76,77,78,79]. PI3K is the kinase responsible for the production of PI(3,4)P_2_ as well as PI(3,4,5)P_3_ [80]. These PIPs function as second messengers for a variety of signaling pathways related to cellular growth, survival and differentiation. PI3K consists of a regulatory subunit, p85, and a catalytic subunit, p110 which phosphorylates PI4P and PI(4,5)P_2_. The molecular mechanism(s) of HCV-mediated PI3K and Akt activation are still not clear, though oxidative stress has been implicated in their activation [81]. HCV NS5A protein has been shown to interact with the PI3K regulatory subunit p85 and release inhibition of catalytic p110 subunit [76,77,82]. The activation of p110 leads to the formation of PI(3,4)P_2_ and PI(3,4,5)P_3_ at the plasma membrane followed by subsequent recruitment of Akt that binds PI(3,4,5)P_3_ or PI(3,4)P_2_ via its PH domain. Upon recruitment to the plasma membrane Akt is activated by Ser 473 phosphorylation by mTORC2 and Thr 308 by phosphoinositide dependent kinase 1 [83,84]. In addition to its role in multiple cellular processes such as glucose metabolism, cell proliferation, transcription, translation and cell migration, Akt also functions as anti-apoptotic protein by inhibiting the pro-apoptotic proteins Bad and Caspase 9 [77,85]. Non-lytic chronic viruses such as Epstein-Barr virus and Polyomavirus that activate PI3K by inhibiting p85 seem to exploit the anti-apoptotic potential of Akt to promote cell survival and maintain a persistent infection. Due to its ability to promote cell survival and block apoptosis, Akt has been implicated in many cancers. An increased oncogenic capacity due to Akt activation in HCV infected cells could play a role in the development of hepatocellular carcinoma. 

The PI3K/Akt pathway is also implicated in promoting *de novo* lipogenesis by activating SREBPs in response to insulin [86]. HCV also stimulates *de novo* lipogenesis by promoting SREBPs activation [87]. The PI3K/Akt pathway could help mediate SREBPs activation in HCV infection. Interestingly, NS4B expression has been shown to promote SREBPs activation and de novo lipogenesis via the PI3K/Akt pathway [88]. PI3K is also known to affect the trafficking of SRBI, which may implicate the kinase in HCV entry, as SRBI is a cellular co-receptor important for HCV entry [89,90,91]. PI3K dependent Akt activation is also regulated by lipid phosphatase and tensin homolog (PTEN), which negates the effect of PI3K and dephosphorylates PI(3,4,5)P_3_ back to PI(4,5)P_2_ thereby disrupting the membrane localization and subsequent activation of Akt [92]. Recently it has been shown that expression of HCV genotype 3a core protein down-regulated PTEN expression by microRNA-dependent blockade of PTEN mRNA translation, leading to the appearance of large lipid droplets [93]. Liver samples from HCV-positive hepatocellular carcinoma patients also displayed reduced PTEN expression [94]. The diverse interactions described indicate a potentially versatile role for PI3K, one that bears further scrutiny in multiple phases of HCV infection.

## 6. Targeting PI4Ks for HCV Therapeutics

The major problem with direct-acting antivirals such as the NS3 inhibitors telaprevir and boceprevir is the emergence of resistant mutants. Antiviral therapies which target host factors may lead to drugs with pan-genotype activity and a greater barrier to resistance. PI4Ks are critical for multiple events in the HCV life cycle, and several other viruses also require intracellular enrichment of PI4P for assembly of viral replication sites, making PI4Ks an excellent target for developing panviral therapeutics. Targeting of host factors is an attractive strategy, but can result in higher toxicity/intolerance, particularly in the case of targets like PI4Ks, which regulate multiple cellular processes. Successful future therapeutics may require selective and targeted inhibition of specific PI4K isoforms to be effective and safe.

AL-9, a member of the 4-anilino quinazoline class of compounds previously considered to be an NS5A inhibitor has been recently shown to inhibit PI4KIIIα and prevent membranous web formation [57]. Pharmacological inhibition of PI4KIIIα with AL-9 resulted in altered subcellular distribution of NS5A reminiscent of that observed upon PI4KIIIα silencing, further confirming that the anti-HCV activity of these compounds involves inhibition of PI4KIIIα and depletion of PI4P from HCV replication sites [57]. New classes of NS5A-binding compounds show promise as extremely potent inhibitors of HCV replication, with EC_50_ values in the picomolar range [95]. While the molecular basis of their inhibition is not clear, it would be interesting to assess the ability of these compounds to interact with NS5A. PIK93, a phenylthiazole has been used to inhibit HCV and Poliovirus replication [51,96]. PIK93 inhibits both the PI4KIII isoforms α and β, but has higher efficacy against the β isoform [97]. The lack of PIK93 specificity for PI4KIII isoforms and the fact that it inhibits PI3Ks at high concentrations raises concern about its potential as an anti-HCV agent [98]. Another potential inhibitor of PI4KIIIα, enviroxime, also displayed strong inhibition of picornaviruses and HCV replication [51,96,99]. More specific PI4KIIIα inhibitors have been identified that potently inhibit replication of HCV genotype 1a and 1b replicons as well as genotype 2a virus [95,100]. 

The PI4KIIIα inhibitors were not cytotoxic in multiple cell lines and primary cell types. However, their anti-proliferative activity in lymphocytes precluded further development, suggesting that intolerance/toxicity to PI4K inhibitors could prove to be an obstacle to their use in the treatment of HCV [100]. Mouse studies to assess the safety of PI4KIIIα inhibitors or knockdown show that homozygotic knockout of the kinase domain or knock-in of a kinase-defective PI4KIIIα displayed a lethal phenotype with widespread mucosal epithelial degeneration of the gastrointestinal tract, though heterozygotes display a less severe phenotype [101]. These observations highlight the pivotal and sensitive role of PI4Ks in cellular physiology, signaling necessary caution in developing PI4K inhibitors for HCV treatment. Abrogation of PI4P signaling by using metabolically stabilized mimetics of PI4P may hold promise for future therapeutic treatments.

## 7. Concluding Remarks

It is known that the both bacteria and viruses exploit phosphoinositide metabolism in various fashions to facilitate their lifecycle. These pathogens rely on remodeling the host cell interior for establishing effective replication platforms. PIPs are pivotal membrane lipid components that help define membrane identity and coordinate a plethora of signaling pathways and interactions at the membrane-cytosol interface. This makes them prime targets for pathogens to harness for creating their necessary unique subcellular environments. Many RNA viruses exploit PI4P and its kinases, and therefore these pathways may have great potential for developing panviral therapeutics, which could prove immensely important as viral recombination continues to drive resistance to current treatments. However, with the myriad functions coordinated by PIPs, targeting PI4P metabolism may present difficulties in the development of potential drugs. The lethality associated with PI4K knockdown and the development of adverse physiological manifestations with PI4K inhibitor treatments suggests that further optimization in required. Our expanding knowledge of uncharacterized viral pathways may shed light on the precise roles of viral proteins in recruiting and activating PI4Ks and PI4P effector proteins to build replication complexes or viral assembly and trafficking hubs. However, with the inherent capacity of RNA viruses to frequently evolve, emergence of resistant mutants that utilize alternate PI4K isoforms or modify the use of PIPs at replication sites is highly probable. 

A number of aspects of the HCV lifecycle are still poorly understood, and several are still subject to vigorous debate. The specific site of viral assembly is still uncharacterized, as is the pathway of its association with VLDL particles during secretion. It is apparent that the viral particle matures while still within the cell, as blocking the Golgi secretory pathway results in increased intracellular infectivity. Like viral assembly, large parts of the maturation process remain to be characterized. As the intricacies of cellular membrane dynamics are further explored, the significance of PIPs in signaling and membrane composition is becoming clearer. The methods cells use to regulate and control protein localizations are also becoming better understood, and PIPs and their associated proteins have begun to gain recognition as significant areas of virion morphogenesis. A deeper insight into how HCV utilizes PIPs and PIP-associated proteins in modulating membranes for HCV maturation/secretion pathways will likely reveal further details of virion co-option of lipoproteins for efficient secretion through the Golgi compartment. The complex interactions outlined here serve to characterize the role of PIPs in the HCV life cycle. Understanding the viral pathways of maturation, morphogenesis, and egress provide unique opportunities for antiviral therapeutics to arrest the infection process.
